# Benzyl 5-hy­droxy-4-oxapenta­cyclo­[5.4.1.0^2,6^.0^3,10^.0^8,11^]dodecane-3-carboxyl­ate

**DOI:** 10.1107/S1600536811009020

**Published:** 2011-03-12

**Authors:** Rajshekhar Karpoormath, Tricia Naicker, Thavendran Govender, Hendrik G. Kruger, Glenn E. M. Maguire

**Affiliations:** a School of Chemistry, University of KwaZulu-Natal, Durban 4000, South Africa; b School of Pharmacy and Pharmacology, University of KwaZulu-Natal, Durban 4000, South Africa

## Abstract

The title compound, C_19_H_18_O_4_, exhibits a long C—C bond [1.575 (2) Å] in the cage structure. In the crystal, pairs of O—H⋯O hydrogen bonds link the mol­ecules into centrosymmetric dimers. C—H⋯O inter­actions also occur.

## Related literature

For examples of PCU cage structures that exhibit C—C bond lengths that deviate from the normal value, see: Flippen-Anderson *et al.* (1991) ▶; Linden *et al.* (2005 ▶). For similar structures, see: Kruger *et al.* (2005 ▶, 2006 ▶); Karpoormath *et al.* (2010 ▶).
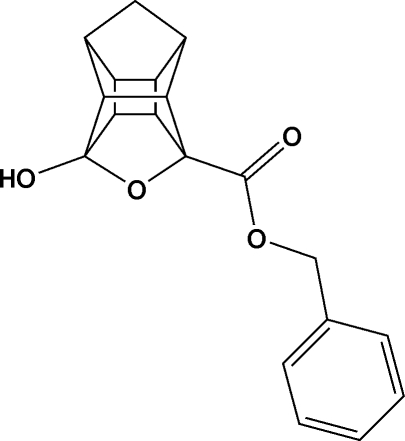


## Experimental

### 

#### Crystal data

C_19_H_18_O_4_
*M* *_r_* = 310.33
Monoclinic, 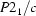

*a* = 6.5254 (2) Å
*b* = 12.8995 (2) Å
*c* = 16.9772 (5) Å
β = 95.243 (1)°
*V* = 1423.07 (6) Å^3^
*Z* = 4
Mo *K*α radiation
μ = 0.10 mm^−1^
*T* = 173 K
0.34 × 0.22 × 0.16 mm

#### Data collection

Nonius KappaCCD diffractometer
6925 measured reflections
3540 independent reflections
2930 reflections with *I* > 2σ(*I*)
*R*_int_ = 0.018

#### Refinement

*R*[*F* ^2^ > 2σ(*F* ^2^)] = 0.048
*wR*(*F* ^2^) = 0.119
*S* = 1.06
3540 reflections
212 parameters
1 restraint
H atoms treated by a mixture of independent and constrained refinement
Δρ_max_ = 0.47 e Å^−3^
Δρ_min_ = −0.24 e Å^−3^

### 

Data collection: *COLLECT* (Nonius, 2000 ▶); cell refinement: *DENZO-SMN* (Otwinowski & Minor, 1997 ▶); data reduction: *DENZO-SMN*; program(s) used to solve structure: *SHELXS97* (Sheldrick, 2008 ▶); program(s) used to refine structure: *SHELXL97* (Sheldrick, 2008 ▶); molecular graphics: *OLEX2* (Dolomanov *et al.*, 2009) ▶; software used to prepare material for publication: *SHELXL97*.

## Supplementary Material

Crystal structure: contains datablocks I, global. DOI: 10.1107/S1600536811009020/is2678sup1.cif

Structure factors: contains datablocks I. DOI: 10.1107/S1600536811009020/is2678Isup2.hkl

Additional supplementary materials:  crystallographic information; 3D view; checkCIF report

## Figures and Tables

**Table 1:** Hydrogen-bond geometry (Å, °)

*D*—H⋯*A*	*D*—H	H⋯*A*	*D*⋯*A*	*D*—H⋯*A*
O1—H1⋯O2^i^	0.97 (2)	1.91 (2)	2.8561 (16)	164 (2)
C3—H3⋯O1^ii^	1.00	2.46	3.3716 (18)	151
C10—H10⋯O3^iii^	1.00	2.41	3.3840 (19)	163

Symmetry codes: (i) 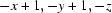; (ii) 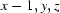; (iii) 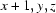.

**Fig. 1.**
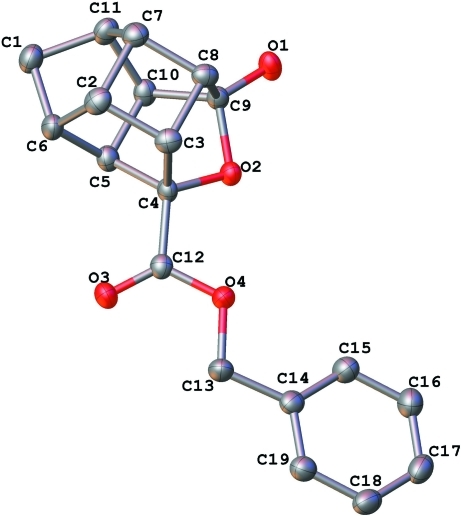
Molecular structure of the title compound, showing the numbering scheme with ellipsoid probability of 50%.

**Fig. 2.**
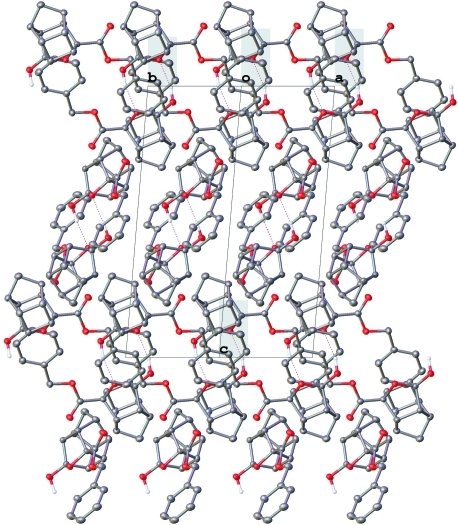
Projection viewed along the [110] with ellipsoid probability of 40%. Only the hydrogen atoms involved in hydrogen bonds are shown. Other hydrogen atoms are omitted for clarity. The hydrogen bonds are shown as dotted lines.

## supplementary crystallographic information

### Comment

We have reported the single-crystal X-ray structure of a range of pentacycloundecane (PCU) derivatives. More recently this included an ether diol (Kruger *et al.*, 2005), a mono-ketone ethyl acetate (Kruger *et al.*, 2006) and a mono-ketone diester (Karpoormath *et al.*, 2010). The tile compound is the first example of a PCU derivative X-ray structure report containing an ether, ester and primary alcohol functional groups (Fig. 1). In the crystal, the ether oxygen hydrogen bonds to the primary alcohol group of its nearest neighbour (O1—H1···O2). This arrangement forms centrosymmetric dimers (Fig. 2).

### Experimental

A mixture of 5-hydroxy-4 oxahexacyclo[5.4.0.0^2,6^.0^3,10^.0^5,9^]dodecane-3-carboxylic acid (1 g, 4.5 mmol), benzyl bromide (6 ml, 4.95 mmol) and K_2_CO_3_ (1.86 g, 13.5 mmol) in DMF (5 ml), was heated to 120 °C and stirred for 5 h. The reaction mixture is cooled to room temperature and diluted with 50 ml of water and stirred further for 10 min. The resulting aqueous reaction mixture was then extracted with dichloromethane (25 ml × 2). The organic layer was dried with anhydrous Na_2_SO_4_, filtered, and concentrated under vacuum. Chromatography with hexane–EtOAc (5:1) afforded products a colourless oil (1.1 g, 79%). Crystallization of the product was carried out by dissolving the pure compound in 2 ml solvent mixture of ethyl acetate and hexane (1:5) and storing the solution at 18 °C (m.p. 397–398 K).

^1^H NMR (CDCl_3_, 400 MHz) δ p.p.m.: 1.55 (1.0H, d, J=10.69 Hz), 1.89 (1.0H, d, J=10.65 Hz), 2.59–2.67 (4.0H, m,), 2.73–2.77 (2.0H, m), 2.97–3.06 (2.0H, m), 4.20 (1.0H, s), 5.19 (2.0H, q, J=11.55 Hz), 7.28–7.33 (5.0H, m).

^13^C NMR (CDCl_3_, 100 MHz) δ p.p.m.: 31.12, 42.10, 42.78, 43.44, 43.52, 46.66, 47.50, 49.88, 57.16, 59.31, 66.82, 89.89, 119.00, 128.24, 128.46, 128.75, 135.77, 171.32, 207.32.

IR (neat) V_max_ cm^-1^: 3430.37, 2961.81, 1739.32, 1453.93, 1336.94, 1267.97, 1199.80, 1137.26, 1091.29, 1078.22, 902.84, 734.29, 692.33, 542.17 cm^-1^.

### Refinement

All the hydrogen atoms, except the hydroxyl hydrogen, were placed in idealized positions in a riding model with *U*_iso_ set at 1.2 or 1.5 times those of their parent atoms and fixed C—H bond lengths. One distance restraint [O1—H1 0.97 (1) Å] was applied.

### Crystal data

**Table:**

C_19_H_18_O_4_	*F*(000) = 656
*M**_r_* = 310.33	*D*_x_ = 1.448 Mg m^−^^3^
Monoclinic, *P*2_1_/*c*	Mo *K*α radiation, λ = 0.71073 Å
Hall symbol: -P 2ybc	Cell parameters from 6932 reflections
*a* = 6.5254 (2) Å	θ = 2.4–28.3°
*b* = 12.8995 (2) Å	µ = 0.10 mm^−^^1^
*c* = 16.9772 (5) Å	*T* = 173 K
β = 95.243 (1)°	Block, colourless
*V* = 1423.07 (6) Å^3^	0.34 × 0.22 × 0.16 mm
*Z* = 4	

### Data collection

**Table:**

Nonius KappaCCD diffractometer	2930 reflections with *I* > 2σ(*I*)
Radiation source: fine-focus sealed tube	*R*_int_ = 0.018
graphite	θ_max_ = 28.3°, θ_min_ = 3.1°
1.2° φ and ω scans	*h* = −8→8
6925 measured reflections	*k* = −17→17
3540 independent reflections	*l* = −22→22

### Refinement

**Table:**

Refinement on *F*^2^	Primary atom site location: structure-invariant direct methods
Least-squares matrix: full	Secondary atom site location: difference Fourier map
*R*[*F*^2^ > 2σ(*F*^2^)] = 0.048	Hydrogen site location: inferred from neighbouring sites
*wR*(*F*^2^) = 0.119	H atoms treated by a mixture of independent and constrained refinement
*S* = 1.06	*w* = 1/[σ^2^(*F*_o_^2^) + (0.0463*P*)^2^ + 0.932*P*] where *P* = (*F*_o_^2^ + 2*F*_c_^2^)/3
3540 reflections	(Δ/σ)_max_ < 0.001
212 parameters	Δρ_max_ = 0.47 e Å^−^^3^
1 restraint	Δρ_min_ = −0.24 e Å^−^^3^

### Special details

**Table:**

Experimental. Half sphere of data collected using *COLLECT* strategy (Nonius, 2000). Crystal to detector distance = 35 mm; combination of φ and ω scans of 1.2°, 30 s per °, 2 iterations.X-ray single-crystal intensity data were collected on a Nonius Kappa-CCD diffractometer using graphite monochromated Mo *K*_α_ radiation (l = 0.71073 Å). Temperature was controlled by an Oxford Cryostream cooling system (Oxford Cryostat). The strategy for the data collections was evaluated using the Bruker Nonius "Collect" program (Nonius, 2000). Data were scaled and reduced using *DENZO-SMN* software (Otwinowski & Minor, 1997). The structure was solved by direct methods and refined employing full-matrix least-squares with the program *SHELXL97* (Sheldrick, 2008) refining on F^2^. The molecular graphics were rendered using OLEX2 (Dolomove *et al.*, 2009). All non-hydrogen atoms were refined anisotropically.
Geometry. All e.s.d.'s (except the e.s.d. in the dihedral angle between two l.s. planes) are estimated using the full covariance matrix. The cell e.s.d.'s are taken into account individually in the estimation of e.s.d.'s in distances, angles and torsion angles; correlations between e.s.d.'s in cell parameters are only used when they are defined by crystal symmetry. An approximate (isotropic) treatment of cell e.s.d.'s is used for estimating e.s.d.'s involving l.s. planes.
Refinement. Refinement of *F*^2^ against ALL reflections. The weighted *R*-factor *wR* and goodness of fit *S* are based on *F*^2^, conventional *R*-factors *R* are based on *F*, with *F* set to zero for negative *F*^2^. The threshold expression of *F*^2^ > σ(*F*^2^) is used only for calculating *R*-factors(gt) *etc*. and is not relevant to the choice of reflections for refinement. *R*-factors based on *F*^2^ are statistically about twice as large as those based on *F*, and *R*- factors based on ALL data will be even larger.

### Fractional atomic coordinates and isotropic or equivalent isotropic displacement parameters (Å^2^)

**Table:**

	*x*	*y*	*z*	*U*_iso_*/*U*_eq_	
O1	0.74558 (16)	0.46393 (9)	0.05935 (6)	0.0284 (3)	
H1	0.695 (4)	0.448 (2)	0.0053 (8)	0.073 (8)*	
O2	0.45389 (15)	0.54964 (8)	0.09726 (6)	0.0223 (2)	
O3	0.06298 (17)	0.60878 (9)	0.22148 (6)	0.0297 (3)	
O4	0.10540 (16)	0.65377 (8)	0.09589 (6)	0.0260 (2)	
C1	0.5554 (3)	0.33580 (14)	0.29940 (9)	0.0311 (4)	
H1A	0.6449	0.3717	0.3411	0.037*	
H1B	0.5203	0.2656	0.3174	0.037*	
C2	0.2721 (2)	0.33660 (12)	0.19839 (9)	0.0279 (3)	
H2	0.1596	0.2865	0.2073	0.034*	
C3	0.2365 (2)	0.41085 (12)	0.12688 (9)	0.0246 (3)	
H3	0.1021	0.4065	0.0937	0.029*	
C4	0.3155 (2)	0.52032 (11)	0.15444 (8)	0.0208 (3)	
C5	0.4540 (2)	0.49471 (12)	0.23047 (9)	0.0244 (3)	
H5	0.4826	0.5553	0.2666	0.029*	
C6	0.3646 (2)	0.39964 (13)	0.27044 (9)	0.0276 (3)	
H6	0.2680	0.4154	0.3113	0.033*	
C7	0.4690 (2)	0.29226 (12)	0.16299 (9)	0.0278 (3)	
H7	0.4703	0.2164	0.1510	0.033*	
C8	0.4308 (2)	0.36722 (12)	0.09197 (9)	0.0250 (3)	
H8	0.4127	0.3363	0.0378	0.030*	
C9	0.5850 (2)	0.45678 (11)	0.10606 (8)	0.0213 (3)	
C10	0.6490 (2)	0.45031 (12)	0.19510 (8)	0.0241 (3)	
H10	0.7798	0.4876	0.2125	0.029*	
C11	0.6469 (2)	0.33477 (13)	0.21925 (9)	0.0284 (3)	
H11	0.7810	0.2975	0.2183	0.034*	
C12	0.1500 (2)	0.59911 (11)	0.16209 (8)	0.0226 (3)	
C13	−0.0640 (2)	0.72567 (13)	0.09816 (9)	0.0286 (3)	
H13A	−0.0465	0.7671	0.1474	0.034*	
H13B	−0.1952	0.6870	0.0976	0.034*	
C14	−0.0697 (2)	0.79630 (12)	0.02758 (9)	0.0250 (3)	
C15	0.0895 (3)	0.80217 (13)	−0.02124 (10)	0.0309 (4)	
H15	0.2077	0.7596	−0.0111	0.037*	
C16	0.0759 (3)	0.87050 (14)	−0.08503 (10)	0.0350 (4)	
H16	0.1843	0.8737	−0.1187	0.042*	
C17	−0.0945 (3)	0.93378 (14)	−0.09964 (10)	0.0347 (4)	
H17	−0.1025	0.9807	−0.1429	0.042*	
C18	−0.2529 (3)	0.92858 (13)	−0.05118 (10)	0.0336 (4)	
H18	−0.3697	0.9722	−0.0610	0.040*	
C19	−0.2416 (3)	0.85979 (13)	0.01181 (10)	0.0296 (3)	
H19	−0.3520	0.8559	0.0445	0.035*	

### Atomic displacement parameters (Å^2^)

**Table:**

	*U*^11^	*U*^22^	*U*^33^	*U*^12^	*U*^13^	*U*^23^
O1	0.0213 (5)	0.0420 (7)	0.0227 (5)	0.0026 (5)	0.0060 (4)	0.0028 (5)
O2	0.0218 (5)	0.0248 (5)	0.0209 (5)	0.0011 (4)	0.0052 (4)	0.0030 (4)
O3	0.0329 (6)	0.0344 (6)	0.0232 (5)	0.0061 (5)	0.0094 (4)	0.0017 (4)
O4	0.0295 (6)	0.0265 (5)	0.0225 (5)	0.0075 (4)	0.0054 (4)	0.0035 (4)
C1	0.0347 (8)	0.0332 (9)	0.0252 (8)	0.0010 (7)	0.0016 (6)	0.0059 (6)
C2	0.0250 (7)	0.0262 (8)	0.0328 (8)	−0.0035 (6)	0.0032 (6)	0.0046 (6)
C3	0.0198 (7)	0.0242 (7)	0.0294 (8)	−0.0018 (6)	0.0010 (6)	−0.0003 (6)
C4	0.0203 (6)	0.0243 (7)	0.0183 (6)	−0.0006 (5)	0.0045 (5)	0.0019 (5)
C5	0.0253 (7)	0.0283 (8)	0.0197 (7)	0.0000 (6)	0.0020 (5)	−0.0005 (6)
C6	0.0307 (8)	0.0303 (8)	0.0227 (7)	0.0045 (6)	0.0071 (6)	0.0054 (6)
C7	0.0303 (8)	0.0230 (7)	0.0304 (8)	0.0009 (6)	0.0042 (6)	0.0002 (6)
C8	0.0230 (7)	0.0248 (7)	0.0269 (7)	0.0018 (6)	−0.0002 (6)	−0.0027 (6)
C9	0.0188 (6)	0.0254 (7)	0.0200 (7)	0.0020 (5)	0.0025 (5)	0.0013 (5)
C10	0.0206 (7)	0.0309 (8)	0.0205 (7)	−0.0015 (6)	0.0005 (5)	0.0013 (6)
C11	0.0253 (7)	0.0331 (8)	0.0263 (8)	0.0033 (6)	−0.0002 (6)	0.0058 (6)
C12	0.0240 (7)	0.0227 (7)	0.0211 (7)	−0.0022 (5)	0.0021 (5)	0.0004 (5)
C13	0.0293 (8)	0.0292 (8)	0.0278 (8)	0.0089 (6)	0.0061 (6)	0.0030 (6)
C14	0.0298 (8)	0.0223 (7)	0.0224 (7)	0.0014 (6)	0.0001 (6)	−0.0028 (6)
C15	0.0325 (8)	0.0296 (8)	0.0310 (8)	0.0041 (7)	0.0047 (7)	0.0038 (6)
C16	0.0420 (10)	0.0348 (9)	0.0290 (8)	0.0017 (7)	0.0077 (7)	0.0036 (7)
C17	0.0498 (10)	0.0285 (8)	0.0250 (8)	0.0022 (7)	−0.0008 (7)	0.0035 (6)
C18	0.0402 (9)	0.0291 (8)	0.0305 (8)	0.0082 (7)	−0.0030 (7)	0.0000 (7)
C19	0.0318 (8)	0.0291 (8)	0.0277 (8)	0.0048 (6)	0.0024 (6)	−0.0019 (6)

### Geometric parameters (Å, °)

**Table:**

O1—C9	1.3740 (17)	C7—C11	1.536 (2)
O1—H1	0.969 (10)	C7—C8	1.548 (2)
O2—C4	1.4355 (16)	C7—H7	1.0000
O2—C9	1.4715 (17)	C8—C9	1.536 (2)
O3—C12	1.2081 (18)	C8—H8	1.0000
O4—C12	1.3362 (18)	C9—C10	1.533 (2)
O4—C13	1.4463 (18)	C10—C11	1.546 (2)
C1—C11	1.535 (2)	C10—H10	1.0000
C1—C6	1.536 (2)	C11—H11	1.0000
C1—H1A	0.9900	C13—C14	1.503 (2)
C1—H1B	0.9900	C13—H13A	0.9900
C2—C6	1.545 (2)	C13—H13B	0.9900
C2—C3	1.547 (2)	C14—C15	1.389 (2)
C2—C7	1.575 (2)	C14—C19	1.395 (2)
C2—H2	1.0000	C15—C16	1.393 (2)
C3—C8	1.553 (2)	C15—H15	0.9500
C3—C4	1.560 (2)	C16—C17	1.384 (3)
C3—H3	1.0000	C16—H16	0.9500
C4—C12	1.497 (2)	C17—C18	1.380 (3)
C4—C5	1.542 (2)	C17—H17	0.9500
C5—C6	1.542 (2)	C18—C19	1.386 (2)
C5—C10	1.565 (2)	C18—H18	0.9500
C5—H5	1.0000	C19—H19	0.9500
C6—H6	1.0000		
			
C9—O1—H1	108.6 (16)	C9—C8—H8	117.7
C4—O2—C9	96.46 (10)	C7—C8—H8	117.7
C12—O4—C13	115.11 (11)	C3—C8—H8	117.7
C11—C1—C6	95.19 (12)	O1—C9—O2	110.72 (11)
C11—C1—H1A	112.7	O1—C9—C10	114.78 (12)
C6—C1—H1A	112.7	O2—C9—C10	104.35 (11)
C11—C1—H1B	112.7	O1—C9—C8	118.94 (12)
C6—C1—H1B	112.7	O2—C9—C8	103.29 (11)
H1A—C1—H1B	110.2	C10—C9—C8	103.19 (12)
C6—C2—C3	108.38 (13)	C9—C10—C11	107.80 (12)
C6—C2—C7	102.71 (12)	C9—C10—C5	101.56 (11)
C3—C2—C7	89.69 (11)	C11—C10—C5	102.95 (12)
C6—C2—H2	117.3	C9—C10—H10	114.4
C3—C2—H2	117.3	C11—C10—H10	114.4
C7—C2—H2	117.3	C5—C10—H10	114.4
C2—C3—C8	90.34 (11)	C1—C11—C7	102.77 (13)
C2—C3—C4	107.64 (12)	C1—C11—C10	103.90 (13)
C8—C3—C4	100.58 (11)	C7—C11—C10	101.75 (12)
C2—C3—H3	118.0	C1—C11—H11	115.5
C8—C3—H3	118.0	C7—C11—H11	115.5
C4—C3—H3	118.0	C10—C11—H11	115.5
O2—C4—C12	112.52 (11)	O3—C12—O4	124.37 (14)
O2—C4—C5	105.42 (11)	O3—C12—C4	122.71 (13)
C12—C4—C5	116.41 (12)	O4—C12—C4	112.88 (12)
O2—C4—C3	104.28 (11)	O4—C13—C14	109.31 (12)
C12—C4—C3	114.78 (12)	O4—C13—H13A	109.8
C5—C4—C3	102.09 (12)	C14—C13—H13A	109.8
C6—C5—C4	108.82 (12)	O4—C13—H13B	109.8
C6—C5—C10	103.38 (12)	C14—C13—H13B	109.8
C4—C5—C10	101.08 (11)	H13A—C13—H13B	108.3
C6—C5—H5	114.1	C15—C14—C19	119.06 (15)
C4—C5—H5	114.1	C15—C14—C13	122.93 (14)
C10—C5—H5	114.1	C19—C14—C13	118.01 (14)
C1—C6—C5	103.84 (12)	C14—C15—C16	120.05 (16)
C1—C6—C2	102.58 (13)	C14—C15—H15	120.0
C5—C6—C2	101.90 (12)	C16—C15—H15	120.0
C1—C6—H6	115.5	C17—C16—C15	120.37 (16)
C5—C6—H6	115.5	C17—C16—H16	119.8
C2—C6—H6	115.5	C15—C16—H16	119.8
C11—C7—C8	108.68 (13)	C18—C17—C16	119.86 (16)
C11—C7—C2	103.28 (12)	C18—C17—H17	120.1
C8—C7—C2	89.51 (11)	C16—C17—H17	120.1
C11—C7—H7	117.1	C17—C18—C19	120.08 (16)
C8—C7—H7	117.1	C17—C18—H18	120.0
C2—C7—H7	117.1	C19—C18—H18	120.0
C9—C8—C7	106.97 (12)	C18—C19—C14	120.57 (16)
C9—C8—C3	102.35 (11)	C18—C19—H19	119.7
C7—C8—C3	90.47 (11)	C14—C19—H19	119.7
			
C6—C2—C3—C8	103.26 (13)	C3—C8—C9—O1	−156.62 (12)
C7—C2—C3—C8	−0.05 (11)	C7—C8—C9—O2	−127.86 (12)
C6—C2—C3—C4	2.05 (16)	C3—C8—C9—O2	−33.53 (13)
C7—C2—C3—C4	−101.26 (12)	C7—C8—C9—C10	−19.38 (14)
C9—O2—C4—C12	−179.39 (12)	C3—C8—C9—C10	74.95 (13)
C9—O2—C4—C5	52.74 (13)	O1—C9—C10—C11	−97.66 (15)
C9—O2—C4—C3	−54.38 (12)	O2—C9—C10—C11	140.99 (12)
C2—C3—C4—O2	127.36 (12)	C8—C9—C10—C11	33.31 (14)
C8—C3—C4—O2	33.59 (13)	O1—C9—C10—C5	154.53 (13)
C2—C3—C4—C12	−109.09 (14)	O2—C9—C10—C5	33.17 (14)
C8—C3—C4—C12	157.15 (12)	C8—C9—C10—C5	−74.51 (13)
C2—C3—C4—C5	17.78 (14)	C6—C5—C10—C9	111.60 (13)
C8—C3—C4—C5	−75.99 (13)	C4—C5—C10—C9	−1.00 (14)
O2—C4—C5—C6	−140.89 (12)	C6—C5—C10—C11	0.06 (14)
C12—C4—C5—C6	93.62 (15)	C4—C5—C10—C11	−112.55 (12)
C3—C4—C5—C6	−32.18 (14)	C6—C1—C11—C7	−53.88 (14)
O2—C4—C5—C10	−32.49 (14)	C6—C1—C11—C10	51.86 (14)
C12—C4—C5—C10	−157.98 (12)	C8—C7—C11—C1	127.80 (13)
C3—C4—C5—C10	76.22 (13)	C2—C7—C11—C1	33.76 (15)
C11—C1—C6—C5	−51.77 (14)	C8—C7—C11—C10	20.41 (15)
C11—C1—C6—C2	54.05 (14)	C2—C7—C11—C10	−73.63 (14)
C4—C5—C6—C1	139.75 (13)	C9—C10—C11—C1	−139.89 (12)
C10—C5—C6—C1	32.92 (14)	C5—C10—C11—C1	−33.04 (14)
C4—C5—C6—C2	33.42 (15)	C9—C10—C11—C7	−33.39 (15)
C10—C5—C6—C2	−73.42 (13)	C5—C10—C11—C7	73.46 (13)
C3—C2—C6—C1	−128.27 (13)	C13—O4—C12—O3	−2.0 (2)
C7—C2—C6—C1	−34.31 (14)	C13—O4—C12—C4	175.61 (12)
C3—C2—C6—C5	−20.96 (15)	O2—C4—C12—O3	−154.75 (14)
C7—C2—C6—C5	73.00 (14)	C5—C4—C12—O3	−32.9 (2)
C6—C2—C7—C11	0.35 (15)	C3—C4—C12—O3	86.21 (18)
C3—C2—C7—C11	109.13 (12)	O2—C4—C12—O4	27.56 (17)
C6—C2—C7—C8	−108.74 (12)	C5—C4—C12—O4	149.38 (13)
C3—C2—C7—C8	0.05 (11)	C3—C4—C12—O4	−91.48 (15)
C11—C7—C8—C9	−0.84 (16)	C12—O4—C13—C14	168.61 (13)
C2—C7—C8—C9	103.02 (12)	O4—C13—C14—C15	−11.6 (2)
C11—C7—C8—C3	−103.91 (13)	O4—C13—C14—C19	169.54 (13)
C2—C7—C8—C3	−0.05 (11)	C19—C14—C15—C16	−0.2 (2)
C2—C3—C8—C9	−107.44 (12)	C13—C14—C15—C16	−178.99 (16)
C4—C3—C8—C9	0.58 (14)	C14—C15—C16—C17	0.8 (3)
C2—C3—C8—C7	0.05 (12)	C15—C16—C17—C18	−0.6 (3)
C4—C3—C8—C7	108.07 (12)	C16—C17—C18—C19	−0.3 (3)
C4—O2—C9—O1	−177.16 (11)	C17—C18—C19—C14	0.9 (3)
C4—O2—C9—C10	−53.15 (12)	C15—C14—C19—C18	−0.6 (2)
C4—O2—C9—C8	54.46 (12)	C13—C14—C19—C18	178.21 (15)
C7—C8—C9—O1	109.05 (14)		

### Hydrogen-bond geometry (Å, °)

**Table:**

*D*—H···*A*	*D*—H	H···*A*	*D*···*A*	*D*—H···*A*
O1—H1···O2^i^	0.97 (2)	1.91 (2)	2.8561 (16)	164 (2)
C3—H3···O1^ii^	1.00	2.46	3.3716 (18)	151
C10—H10···O3^iii^	1.00	2.41	3.3840 (19)	163

Symmetry codes: (i) −*x*+1, −*y*+1, −*z*; (ii) *x*−1, *y*, *z*; (iii) *x*+1, *y*, *z*.
